# S26. HERITABILITY OF SOCIAL MISTRUST IN CHILD AND ADOLESCENT NON-CLINICAL SAMPLES: A HEALTHY TWINS STUDY

**DOI:** 10.1093/schbul/sby018.813

**Published:** 2018-04-01

**Authors:** Han-yu Zhou, Keri Wong, Li-juan Shi, Xi-long Cui, Yun Qian, Ya-song Du, Simon S Y Lui, Xue-rong Luo, Eric F C Cheung, Anna Docherty, Raymond Chan

**Affiliations:** 1 Institute of Psychology; 2 University of Cambridge; 3 Hunan University of Science and Technology; 4 Central South University; 5 Shanghai Mental Health Centre; 6 Castle Peak Hospital; 7 VIPBG

## Abstract

**Background:**

Paranoia, or excessive suspiciousness of others, has been one of the core psychotic symptoms of schizophrenia. Recent studies have extended the study of psychotic symptoms in clinical groups to psychotic-like experiences in the general population. Few studies have systematically examined the prevalence of paranoid thinking or its attenuated form, social mistrust, in young children in the community. The present study examined the Social Mistrust Scale (SMS) and utilized it to examine the structure, prevalence, and heritability of social mistrust in a large sample of Chinese children and adolescents.

**Methods:**

We administered the SMS to 1047 pairs of healthy twins aged 8 to 14 years and conducted structural equation modelling (SEM) to assess the structure of the SMS. Heritability of social mistrust was estimated in a sub-sample of twins (n=959 pairs). Finally, we examined administered the SMS to 32 adolescents with childhood-onset schizophrenia and 34 healthy controls to examine the convergent validity between the SMS and the Positive and Negative Syndrome Scale (PANSS).

**Results:**

The SEM showed a three-factor structure for social mistrust (home, school, and general mistrust). Social mistrust was moderately heritable (39%, 95% CI [21%-59%]) with context-dependent sex differences. The SMS exhibited good discriminant validity in distinguishing adolescents with childhood-onset schizophrenia from healthy controls (AUC=0.80), and good convergent validity with the Positive and Negative Syndrome Scale (rs = 0.33–0.45).

**Discussion:**

Taken together, the present findings showed a stable latent structure of the SMS in a large-scale non-clinical sample of children and adolescents. We found a moderate heritability estimate for social mistrust (39%) in a large healthy-twin sample. In addition, significant gender differences were found, where home mistrust was heritable for males (58%) but not for females, and school mistrust was heritable for females (54%) but not for males. Finally, we also demonstrated that the SMS possesses good discriminate validity in identifying adolescents with childhood-onset schizophrenia from healthy controls and convergent validity with standardized clinical measures of schizophrenia symptoms.

